# When personality gets under the skin: Need for uniqueness and body modifications

**DOI:** 10.1371/journal.pone.0245158

**Published:** 2021-03-03

**Authors:** Selina M. Weiler, Bjarn-Ove Tetzlaff, Philipp Yorck Herzberg, Thomas Jacobsen

**Affiliations:** 1 Experimental Psychology Unit, Helmut Schmidt University/University of the Federal Armed Forces Hamburg, Hamburg, Germany; 2 Personality Psychology and Psychological Assessment, Faculty of Humanities and Social Sciences, Helmut Schmidt University of the Federal Armed Forces, Hamburg, Germany; Universitat Wien, AUSTRIA

## Abstract

Do individuals modify their bodies in order to be unique? The present study sought to investigate need for uniqueness (NfU) subcomponents as possible motives for modifying one’s body. To this end, the study obtained information from 312 participants about their NfU (using the German NfU-G global scale and three sub-scales) and their body modifications (tattoos, piercings, and extreme body modifications such as tongue splitting). By analyzing the three subcomponents of NfU, the study was able to investigate the differential relationship of the sub-scales with the outcome measures, which facilitated a fine-grained understanding of the NfU–body-modification relationship. The study found that tattooed, pierced, and extreme-body-modified individuals had higher NfU-G scores than individuals without body modifications. Moreover, it seemed that individuals with tattoos took a social component into consideration while lacking concern regarding others’ reaction toward their tattoos, although not wanting to cause affront. Pierced and extreme-body-modified individuals, contrarily, tended to display a propensity to actively flout rules and not worry about others’ opinions on their modifications. However, although statistically significant, the effect size (*d*) for the NfU-G differences in the tattooed and pierced participants’ mean scores was small to medium in all three subcomponents. The extreme-body-modified group presented medium and medium to large effects. Further, the study observed that the number of body modifications increased with an increasing NfU in tattooed and pierced individuals. These findings demonstrated multifaceted interrelations between the NfU, its subcomponents, and the three kinds of body modifications investigated in the present study.

## Introduction

Body modifications of all kinds, especially tattoos and piercings, are no longer a rare phenomenon. There has been a steady increase in the number of body-modified individuals in Western countries [1–3]. However, the trend is flattening out due to the increasing popularity of modifying the body [4–9]. Individuals now resort to even more radical body modifications. Extreme and risky procedures, such as implanting objects under the skin, splitting the tongue, or inducing skull deformity, are becoming steadily more popular [10, 11]. In the case of several procedures, high health risks, long healing processes, social stigmatization, pain, permanent changes of the body, and monetary costs must be taken into account [12–15]. This applies, for example, to tattoos—the selective puncturing of the skin with needles through which dyes are injected into the skin’s middle layer (the dermis; [16]), piercings—involving the piercing of a channel through the skin with a cannula so that a piece of jewelry can be inserted [11]—and extreme body modifications—associated with higher psychological and physical risks (e.g., subdermal implants, scarification, tongue splitting) [11, 17, 18].

The most widespread and primary motives for tattooing and piercing seem to be self-expression, a sense of identity, and uniqueness [e.g., 6, 15, 19–25]. Other motives that found previous studies have found are beautifying one’s own body and generating or augmenting individuality or individual symbols, indicating group affiliation, and several others [e.g., 4, 5, 7, 16, 26–37]. Wohlrab and colleagues found a total of 10 motivational domains in their review [26].

Several studies considered the desire to differ from others—need for uniqueness (NfU)—as a relatively stable personality trait [38, 39]; Snyder and Fromkin’s [39] “Theory of Uniqueness” provides its theoretical framework. NfU can be understood as a motivation for counteracting temporarily prevailing situational conditions, for example, when an individual has an impression of being too similar to or too dissimilar from others [38–40]. It is characterized by a willingness to violate social conventions and a tendency to behave in a nonconformist manner [38, 41]. Several studies concerning the interrelation between body modifications and NfU, mainly concerning tattoos and piercings presenting either small to moderate [e.g., 15, 42, 43] or moderate to large effect sizes [e.g., 20], already exist. They found a significantly higher expression of NfU among tattooed than non-tattooed individuals [15, 19, 20, 42–45]. However, individuals with a high NfU are more likely to have their bodies modified than those without [46].

Nevertheless, the relation between NfU and extreme body modifications has not been investigated yet. The spread of body modifications [1–3, 47] leads to the assumption that having a body modification loses its weight in society. Individuals have to engage in even more unique body modifications, which may be extreme and even more invasive [48]. Engaging in such extreme procedures may be associated with an even higher NfU, as individuals have to hazard the consequences of possible psychological and physiological risks [11, 17, 18].

The present study addressed this issue by considering three types of body modifications (piercings, tattoos, and extreme body modifications) separately using the novel German-adapted NfU-G scale [49]. It adapted items to fit the German-speaking population, eliminating items based on cultural differences between the United States and Germany [49]. Snyder and Fromkin [38] extracted three sub-scales by exploring the Uniqueness Scale’s internal structure, indicating that subcomponents contributed to the global NfU. The first sub-scale, Factor 1, reflects “a lack of concerns regarding others’ reactions to one’s different ideas, actions, and so on”^38(p.522-523)^. The second sub-scale, Factor 2, reflects “a person’s desire to not always follow rules”^38(p.523)^. The third sub-scale, Factor 3, reflects “a person’s willingness to publicly defend his or her beliefs” ^38(p.523)^. Snyder and Fromkin’s [38, 39] validation studies were conducted only for the global scale; therefore, this study applied the NfU-G.

Focusing on the three subcomponents of NfU individually allows the investigation of the differential relationship of the outcome measures’ sub-scales, which may facilitate a fine-grained understanding of the NfU–body-modification relation. NfU, being a multidimensional construct, may show how individuals having different types of body modifications differ with respect to different subcomponents of NfU. Assuming individuals acquire body modifications in the absence of being motivated by, for example, rebellion [6, 19], which are well planned [25] and serve as beautification [26], we can expect differences in the sub-scales’ results.

## Materials and methods

### Participants

An a priori power analysis using G*Power 3.1 [50] was performed for sample size estimation. With an alpha = .05 and Cohen’s *d* = 0.50 [51], the projected sample size needed with this effect size is approximately N = 176 for the between-group comparisons to uncover medium-sized effects. Thus, our proposed total sample size of 312 was more than adequate for this study’s primary objective, allowing for expected attrition, and our additional objectives of controlling for possible subgroup analysis.

A group of 312 individuals (194 females, 117 males, and one nonbinary) aged between 18 and 66 years (*M* = 28.58, *SD* = 11.57) volunteered to participate in this study (S1 Table). Since we recorded data from human individuals, we obtained informed consent from each participant by online affirmation. Nonetheless, the study was conducted anonymously. Furthermore, we performed the study was performed according to the Declaration of Helsinki and ensured its approval by the psychological ethics review board of the Helmut Schmidt University/University of the Federal Armed Forces Hamburg. Only individuals residing in Germany at the time of the survey participated in the study. Most participants were students (46.15%, *Mdn* = 4.00, IQR = 3) and single (47.12%, *Mdn* = 2.00, IQR = 2) and declared their highest academic qualification as having been in school for at least 12 years (51.92%, *Mdn* = 4.00, IQR = 1) or a completed Abitur (a diploma from German secondary school qualifying individuals for university admission). Moreover, 138 (44.24%) participants stated that they were body-modified (*Mdn* = .00, IQR = 1). Among them 33% (*Mdn* = 2.00, IQR = 1) were tattooed, 33.3% (*Mdn* = 2.00, IQR = 1) were pierced, and 8% (*Mdn* = 2.00, IQR = 0) were extreme-body-modified. Further, 174 (55.77%) participants reported to having no body modification at all. Using a Tetrachoric correlation, we observed that being tattooed and pierced correlated largely (*r*_*tet*_*(310)* = .74, 95% CI [.68, .78], *p* < .001). Similarly, being tattooed and extreme-body-modified correlated largely (*r*_*tet*_*(310)* = .71, 95% CI [.65, .76], *p* < .001). Being pierced and extreme-body-modified also correlated largely (*r*_*tet*_*(310)* = .76, 95% CI [.71, .81], *p* < .001). Among all types of body modifications, 92.75% were visible (*Mdn* = 1.00, IQR = 0). The participants reported having between 1 and 48 tattoos (*M* = 5.13, *SD* = 6.78, *Mdn* = 3.00, IQR = 5), between 1 and 28 piercings (*M* = 3.34, *SD* = 3.61, *Mdn* = 2.00, IQR = 3), and between 1 and 5 extreme body modifications (*M* = 2.08, *SD* = 1.00, *Mdn* = 2.00, IQR = 1). The tattooed participants reported having between 1% and 80% of their bodies covered with tattoos (*M* = 10.50, *SD* = 13.55, *Mdn* = 5.00, IQR = 13).

### Materials and procedure

The entire study was conducted online via the online survey tool Unipark (https://www.unipark.com/). The online questionnaire link was posted on forums focusing on body modification and social media feeds (S1 Forums). Posters and flyers were displayed at the university and its library and clubhouse. Moreover, the link was sent via e-mail to students and university staff and via messenger apps such as WhatsApp to friends and associates over a total period of 3 months.

The study was divided into three sections. The first two sections appeared in a randomized order. One of these two sections consisted of the translated version of the English Need for Uniqueness scale, the German NfU-G scale [49], a self-report questionnaire used to measure individual participants’ NfU. On a five-point Likert scale (“totally agree” to “totally disagree”), the participants assessed how they perceived their characteristics and behaviors in certain situations. The German version of the scale comprised 26 items, assessing the global NfU and the subcomponents, as measured by items of Factors 1, 2, and 3. Factor 1 included items such as “I find that criticism affects my self-esteem”^49(p. 237)^, Factor 2, items such as “I find it sometimes amusing to upset the dignity of teachers, judges, and ‘cultured’ people”^49(p.237)^, and Factor 3, items such as “When I am in a group of strangers, I am not reluctant to express my opinion publicly” ^49(p.237)^. In the present study, McDonald’s Omega for the NfU-G global score was *ω* = .82. For Factor 1, *ω* = .81, for Factor 2, *ω* = .65, and for Factor 3, *ω* = .70.

In the other one of the first two sections, the participants were asked to answer questions about their body modifications, indicating whether tattoos, removed tattoos, piercings (except in the first ear lobe; [19]), or extreme body modifications (e.g., cutting, tunnel, tongue splitting, etc.) were present at all. If all these items were negated, the study ended for the participants of the comparison group. If the answer to at least one of the questions was “yes,” additional questions for the respective existing body modifications were displayed. For each type of modification, it was asked whether at least one of these was visible when the participant wore summer clothing. Moreover, for each type of body modification other than removed tattoos, participants were asked how many modifications they had undergone. Tattooed participants were also given a question item regarding the body’s estimated area covered by tattoos; the estimated value was given as a percentage.

In the last part of the questionnaire, all participants were asked to answer sociodemographic questions. This part included question items on age, gender, residence and birth, marital status, highest educational level, current employment, chronic physical illnesses, psychiatric illnesses, existing traumas, tobacco, alcohol consumption, and use of illegal drugs.

## Results

Initial analyses showed that tattooed participants differed by age (*X*^*2*^[4] = 26.74, *p* < .001, φ_c_ = .29, a medium effect [51]), suggesting that the participants aged between 26 and 35 (*z* = 3.87, *p* < .001) had significantly more tattoos than those between 46 and 55 (*z* = 3.21, *p* < .001), by gender (*X*^*2*^[2] = 18.65, *p* < .001, φ_c_ = .24, a small to medium effect [51]), suggesting that women (*z* = 3.96, *p* < .001) had significantly more tattoos than men (*z* = -4.13, *p* < .001), but not by educational level (*X*^*2*^[5] = 8.57, *p* = .13, φ_c_ = .17). Pierced participants significantly differed by age (*X*^*2*^[4] = 32.93, *p* < .001, φ_c_ = .33, medium effect [51]), suggesting that the participants aged between 26 and 35 (*z* = 4.14, *p* < .001) had significantly more piercings than those between the age of 46 and 55 (*z* = -3.95, *p* < .001), by gender (*X*^*2*^[2] = 60.30, *p* < .001, φ_c_ = .44, a medium to large effect [51]), suggesting that women (*z* = 7.51, *p* < .001) had more piercings than men (*z* = -7.69, *p* < .001) but not by educational level (*X*^*2*^[5] = 6.63, *p* = .25, φ_c_ = .15). Extreme-body-modified participants did not differ by age (*X*^*2*^[4] = 7.43, *p* = .12, φ_c_ = .14) but differed by gender (*X*^*2*^[2] = 16.22, *p* < .001, φ_c_ = .23, a small to medium effect [51]), suggesting that nonbinary individuals (*z* = 3.39, *p* < .001) had significantly more extreme body modifications. However, they did not differ by educational level (*X*^*2*^[5] = 5.36, *p* = .37, φ_c_ = .13 [51]).

Unpaired t-tests detected differences in the NfU-G global scale ratings and the three factors between tattooed, pierced, and extreme-body-modified participants. All included tests were subjected to a Bonferroni correction (S1 File) to counteract the cumulative Type-I error [52]. Alpha value was adjusted to *p* = .0125.

The results (Table 1) showed a significant difference between the tattooed group (*M* = 3.40, *SD* = .45, *n* = 103) and the non-tattooed group (*M* = 3.20, *SD* = .43, *n* = 209), *t*(310) = 3.64, *p* < .001, with a small to medium effect (*d* = 0.44; 95% CI [.09, .29]; [51]) for the NfU-G global score, and between the tattooed group (*M* = 3.72, *SD* = .55, *n* = 103) and non-tattooed group (*M* = 3.49, *SD* = .57, *n* = 209) for Factor 1, “lack of concern,” *t*(310) = 3.31, *p* < .001, with a small to medium effect (*d* = 0.40, 95% CI [.09, .36]; [51]). However, Factor 2, “not follow rules,” was found to be nonsignificant between the tattooed group (*M* = 3.04, *SD* = .60, *n* = 103) and the non-tattooed group (*M* = 2.87, *SD* = .55, *n* = 209), *t*(310) = 2.42, *p* = .02, and *d* = 0.29 (95% CI [.03, .30]; [51]). For Factor 3, “defend beliefs publicly,” between the tattooed group (*M* = 3.23, *SD* = .72, *n* = 103) and the non-tattooed group (*M* = 3.07, *SD* = .65, *n* = 209), *t*(310) = 1.92, *p* = .06, and *d* = 0.23 (95% CI [-.004, .32]; [51]), nonsignificant results were found.

**Table 1:** Tattooed and non-tattooed group comparison using unpaired *t-*test.

Variable	Tattoo	*n*	*M*	*SD*	*t*	*p*	*Cohen’s d*	*95% CI*
Global score	Yes	103	3.40	.45	3.64	.001**	0.44	.09, .29
	No	209	3.20	.43				
Lack of concern	Yes	103	3.72	.55	3.31	.001**	0.40	.09, .36
	No	209	3.49	.58				
Not follow rules	Yes	103	3.04	.60	2.42	.02	0.29	.03, .30
	No	209	2.87	.55				
Defend beliefs publicly	Yes	103	3.23	.72	1.92	.06	0.23	-.004, .32
	No	209	3.07	.65				

*n*, subsample size; *M*, means; *SD*, standard deviation; *t*, *t*-test

**, *p ≤* .001; CI, confidence interval.

In a succeeding step, the results (Table 2) showed a significant difference between the pierced (*M* = 3.37, *SD* = .43, *n* = 104) and non-pierced group (*M* = 3.22, *SD* = .45, *n* = 208) for the NfU-G global score, *t*(310) = 2.77, *p* = .01, with a small to medium effect (*d* = 0.33; 95% CI [.04, .25]; [51]). No group difference was found between the pierced (*M* = 3.63, *SD* = .55, *n* = 104) and the non-pierced group (*M* = 3.53, *SD* = .59, *n* = 208) for Factor 1, “lack of concern,” *t*(310) = 1.42, *p* = .16, and *d* = 0.17, 95% CI [-.04, .23]. Factor 2, “not follow rules,” was found to be significant between the pierced (*M* = 3.10, *SD* = .62, *n* = 104) and the non-pierced group (*M* = 2.84, *SD* = .53, *n* = 208), *t*(310) = 3.94, *p* < .001. The effect size (*d* = 0.47; 95% CI [.13, .40]) corresponds to a small to medium effect [51]. Factor 3, “defend beliefs publicly,” was found to be nonsignificant between the pierced (*M* = 3.18, *SD* = .68, *n* = 104) and the non-pierced group (*M* = 3.10, *SD* = .68, *n* = 208), *t*(310) = 1.06, *p* = .29, and *d* = 0.13 (95% CI [-.07, .25]; [51]).

**Table 2:** Pierced and non-pierced group comparison using unpaired *t-*test.

Variable	Piercing	*n*	*M*	*SD*	*t*	*p*	*Cohen’s d*	*95% CI*
Global score	Yes	104	3.37	.43	2.77	.01*	0.33	.04, .25
	No	208	3.22	.45				
Lack of concern	Yes	104	3.63	.55	1.42	.16	0.17	-.04, .23
	No	208	3.53	.59				
Not follow rules	Yes	104	3.10	.62	3.94	.001**	0.47	.13, .40
	No	208	2.84	.53				
Defend beliefs publicly	Yes	104	3.18	.68	1.06	.29	0.13	-.07, .25
	No	208	3.10	.68				

*n*, subsample size; *M*, means; *SD*, standard deviation; *t*, t-test

*, *p* ≤ .01

**, *p ≤* .001; CI, confidence interval.

Same analysis was applied for the extreme-body-modified group (Table 3). The results showed significant differences between the extreme-body-modified (*M* = 3.50, *SD* = .33, *n* = 25) and the non-extreme-body-modified group (*M* = 3.25, *SD* = .45 *n* = 287) for the NfU-G global score, *t*(310) = 2.79, *p* = .01, with a medium effect (*d* = 0.58; 95% CI [.08, .44]; [51]). The study revealed no group difference between the extreme-body-modified (*M* = 3.67, *SD* = .50 *n* = 25) and the non-extreme-body-modified group (*M* = 3.56, *SD* = .58 *n* = 287) was found regarding Factor 1, “lack of concern,” *t*(310) = 0.97, *p* = .33, and *d* = 0.20 (95% CI [-.12, .35]). Factor 2, “not follow rules,” was significant between the extreme-body-modified (*M* = 3.40, *SD* = .56, *n* = 25) and the non-extreme-body-modified group (*M* = 2.89, *SD* = .56, *n* = 287), *t*(310) = 4.43, *p* < .001. The effect size (*d* = 0.92; 95% CI [.29, .74]) indicated a large effect [51]. Regarding Factor 3, “defend beliefs publicly,” the results were nonsignificant between the extreme-body-modified (*M* = 3.30, *SD* = .57, *n* = 25) and the non-extreme-body-modified group (*M* = 3.11, *SD* = .69, *n* = 287), *t*(310) = 1.35, *p* = .18, and *d* = 0.28; 95% CI [-.09, .47]; [51]).

**Table 3:** Extreme-body-modified and non-extreme-body-modified group comparison using unpaired *t-*test.

Variable	Extreme body modification	*n*	*M*	*SD*	*t*	*p*	*Cohen’s d*	*95% CI*
Global score	Yes	25	3.50	.33	2.79	.01*	0.58	.08, .44
	No	287	3.25	.45				
Lack of concern	Yes	25	3.67	.50	0.97	.33	0.20	-.12, .35
	No	287	3.56	.58				
Not follow rules	Yes	25	3.40	.56	4.43	.001**	0.92	.29, .74
	No	287	2.89	.56				
Defend beliefs publicly	Yes	25	3.30	.57	1.35	.18	0.28	-.09, .47
	No	287	3.11	.69				

*n*, subsample size; *M*, means; *SD*, standard deviation; *t*, t-test

*, *p* ≤ .01

**, *p ≤* .001; CI, confidence interval.

A negative binomial regression was used to test whether the number of body modifications in total, tattoos, piercings, and extreme body modifications, varied depending on the scores of the NfU-G. On increasing the NfU-G global score by one scale point, the number of body modifications increased significantly by the factor exp(*b*) = 2.88 (*b* = 1.06, *p* < .001, *n* = 138, 95% Wald CI [1.85, 4.48]), the number of tattoos increased significantly by the factor exp(*b*) = 2.95 (*b* = 1.08, *p* < .001, *n* = 103, 95% Wald CI [1.75, 4.98]), and the number of piercings increased significantly by the factor exp(*b*) = 3.21 (*b* = 1.17, *p* < .001, *n* = 104, 95% Wald CI [1.90, 5.42]), whereas the number of extreme body modifications did not increase significantly (*b* = .80, *p* = .22, *n* = 25, 95% Wald CI [0.62, 8.04]). When the value of Factor 1, “lack of concern,” increased by one scale point, the number of body modifications increased significantly by the factor exp(*b*) = 1.73 (*b* = .55, *p* = .002, *n* = 138, 95% Wald CI [1.23, 2.45]), the number of tattoos increased significantly by the factor exp(*b*) = 1.72 (*b* = .54, *p* = .02, *n* = 103, 95% Wald CI [1.11, 2.65]), and the number of piercings increased significantly by the factor exp(*b*) = 1.94 (*b* = .66, *p* < .001, *n* = 104, 95% Wald CI [1.30, 2.90]), whereas the number of extreme body modifications did not increase significantly (*b* = .37, *p* = .45, *n* = 25, 95% Wald CI [0.55, 3.77]). Increasing Factor 2, “not follow rules,” by one scale point increased significantly the number of body modifications by the factor exp(*b*) = 1.69 (*b* = .53, *p* = .002, *n* = 138, 95% Wald CI [1.22, 2.34]), the number of tattoos by the factor exp(*b*) = 1.85 (*b* = .61, *p* = .002, *n* = 103, 95% Wald CI [1.26, 2.70]), and the number of piercings by the factor exp(*b*) = 1.55 (*b* = .44, *p* = .02, *n* = 104, 95% Wald CI [1.07, 2.23]), but not the number of extreme body modifications (*b* = .32, *p* = .40, *n* = 25, 95% Wald CI [0.65, 2.88]). Increasing Factor 3, “defend beliefs publicly,” increased significantly the number of body modifications by the factor exp(*b*) = 1.58 (*b* = .46, *p* < .001, *n* = 138, 95% Wald CI [1.22, 2.06]), the number of tattoos by the factor exp(*b*) = 1.53 (*b* = .43, *p* = .01, *n* = 103, 95% Wald CI [1.13, 2.09]), and the number of piercings by the factor exp(*b*) = 1.65 (*b* = .50, *p* < .001, *n* = 104, 95% Wald CI [1.22, 2.24]), whereas, again, the number of extreme body modifications did not increase significantly (*b* = .23, *p* = .49, *n* = 25, 95% Wald CI [0.66, 2.43]).

To detect possible confounding variables, a multiple linear regression analysis was computed. Neither age and gender nor education had an influence on the NfU-G global score (*F*[3, 308] = 1.22, *p* = .30) with an *R*^*2*^ = .01 (*f*^*2*^ = .01), the Factor, 1 “lack of concern,” (*F*[3, 308] = .29, *p* = .83) with an *R*^*2*^ = .003 (*f*^*2*^ = .003), and the Factor 3, “defend beliefs publicly,” (*F*[3, 308] = 1.60, *p* = .19) with an *R*^*2*^ = .02 (*f*^*2*^ = .02). This study found that age explained a significant amount of the variance of Factor 2, “not follow rules,” (*F*[3, 308] = 5.01, *p* = .002) with an *R*^*2*^ = .05 (*f*^*2*^ = .05), indicating that age could have significantly predicted Factor 3 (β = -.19, *p* < .001) with a small effect [53].

A post-hoc power analysis was performed using the specification of Cohen’s effect size *d* = 0.5 [51] to determine achieved power for each group. It revealed a power (1 – β err prob) of .99 for the tattooed/non-tattooed groups and the pierced/non-pierced groups and .67 for the extreme-body-modified/non-extreme-body-modified groups.

## Discussion

Investigating differences between mainly student participants’ NfU and body modifications, the study demonstrated multifaceted interrelations between the NfU, its subcomponents, and the three kinds of body modifications investigated in the present study.

We began by examining whether tattooed, pierced, and extreme-body-modified individuals differ from individuals without any body modifications regarding their expression on the German NfU-G scale. The results indicate that tattooed, pierced, and extreme-body-modified participants scored significantly higher than non-tattooed, non-pierced, and non-extreme-body-modified participants with small to large effect sizes. These results align with previous literature [e.g., 19, 20, 43], contributing with the findings on extreme body modifications.

Regarding the three types of modifications and subcomponents individually, Factors 1, the “lack of concern regarding others’ reactions to one’s different ideas, actions, and so on” ^38(p.522-523)^, and 2, “a person’s desire to not always follow rules” ^38(p.522)^, were found significant.

Interestingly, and supporting previous assumptions, the extreme-body-modified participants presented the greatest effects in both the NfU-G global score and the significant Factor 2 across body modification groups. It seems as if taking this further step in undergoing an even more invasive body modification [48] suggests a higher NfU.

Regarding the differences between the tattooed and non-tattooed groups, only the NfU-G global score and Factor 1, “lack of concern,” were significant with small to medium effect sizes. The lack of concern regarding others’ reactions to one’s different ideas, actions, and so on was found to be related to more extraverted individuals who are emotionally stable and worry less about others’ reactions and perceptions [49]. This supports the idea that tattooed individuals have the urge to express their uniqueness in a social context, which does not necessarily intend to affront others in doing so [49]. One may assume that tattooed individuals, scoring higher than non-tattooed individuals, may use tattoos as a social aspect of their lives. As tattoos are becoming increasingly popular, acquiring a tattoo is not related to not wanting to obey rules, as there are no rules anymore that could dissuade someone from undergoing the procedure of having a tattoo or risking social exclusion, or an urge to express their uniqueness in defending their beliefs publicly and openly. Individuals not following the rules may not respect the norms of modesty and politeness, which may be connected to wanting to publicly defend these beliefs [54], resulting in social stigmatization [55]. This seems to be unwanted by tattooed individuals.

On the other hand, the pierced, non-pierced, and the extreme-body-modified and non-extreme-body-modified groups were significantly different on the NfU-G global and Factor 2, “not follow rules,” scores with small to large effects. The desire to not always follow rules was found to have a lesser social component while illustrating the willingness to take some risks and seek stimulation [49]. Individuals are more open to new experiences and are less agreeable and conscientious [49]. Schumpe and colleagues [49] postulate that there may be some kind of motivated reasoning [56] being present and, in so, individuals believe there will be no negative consequences from one’s actions pursuing nonconforming behavior. Considering inserting a piece of jewelry in the skin with a cannula through a channel [11] or extreme body modifications associated with higher psychological and physical risks [11, 17, 18] seems to suggest a lack of social conformity and classic stereotypes for body modifications, albeit previous studies indicate the opposite [e.g., 25]. It is assumable that these findings could be based on the fact that participants having the desire to not constantly follow rules could be understood, as mentioned earlier, as not respecting norms of modesty and politeness [54].

The willingness to defend one’s beliefs publicly, on the other hand, was found nonsignificant in all three types of body modification. Being less concerned about the evaluation of others is an essential requirement for this Factor [49]. Assuming it is not present in tattooed, pierced, or extreme-body-modified individuals, concern about social disapproval is implied. The willingness to speak up for one’s opinion seems to not be a central aspect in acquiring at least one of the three body modifications in a mainly student sample. However, acquiring a body modification without having to fear social disapproval would require stepping out and opposing the majority’s opinion to enable social change [41], since modifying one’s own body has long been socially taboo in Germany. It is still not fully socially accepted [21].

Despite this study’s significant results, small to medium effects were observed. The most evident conclusion is that these effects may be a result of tattooed and non-tattooed individuals being more similar than different [57], in our case, relating to tattooed, pierced, and extreme-body-modified individuals. The assumption of group differences in the expression of NfU is based on the premise that body modifications are sufficient to make an individual appear unique. Moderation of the effect by another factor, such as one of the many other motives [4, 5, 7, 16, 21–24, 26–37, 58–61], could have contributed to the profound effects. However, non-body-modified individuals also have a high NfU that is not reflected in undergoing body modifications but rather in other behaviors, such as exceptional consumer behavior [46].

Of course, for NfU to become manifest in overt behavior, i.e., to result in actual body modification, the given person–situation interaction, in a field-theoretical sense [62], must allow it. Lewin viewed behavior as a function of the individual and the environment [62]. If the given situation or the social context in a broader sense is strong enough, either the majority will undergo body modification, or nobody will. In very strong situations, NfU ceases to matter. Moreover, some individuals may have benefitted from an increasingly de-tabooed education to stand out and accept their individuality [63, 64] or had the social and legal possibility of free personal development [21, 65], resulting in possible body modifications and a higher NfU, while others may have not.

Further, we investigated whether the number of body modifications increased depending on individuals’ NfU. The results indicated a significant positive influence of NfU on the number of body modifications, tattoos, and piercings, bearing out findings of Tiggemann and Hopkins [20]. A distinct NfU, which implies a certain resistance to the opinion of others regarding one’s actions, having the desire not to follow rules, and being prepared to represent one’s attitudes to others publicly, could consequently promote the modification of one’s body to resist the social stigmatization that results from it, as body modifications are increasingly gaining social acceptance [55]. To maintain the expression of nonconformity and uniqueness, an individual could counteract this tendency with further modifications, again unusual and unique ones, in order to remain within the social taboo [55]. Nonetheless, the findings did not apply to the extreme-body-modified group. It appears that having a higher score on the NfU-G scale does not entail undergoing more extreme body modifications. This may be due to the fact that extreme body modifications are not as prominent as tattoos and piercings. Moreover, there are limited possibilities for undergoing surgical procedures in Germany. Besides, only one participant had extreme body modifications in the absence of tattoos or piercings.

Nevertheless, we encourage our results to be viewed critically. Since the study was an online survey, the possibility of survey fraud, influencing external factors, motivation, circadian timing, or the influencing environment could not be ruled out [66]. Nevertheless, it could have increased the feeling of anonymity and reduced social desirability bias [67], counteracting the Rosenthal effect [68, 69], and the standardization could have ensured a high degree of objectivity, as both the survey and the evaluation were computer-based [67]. Likewise, familiarity with the diagnostic tool cannot be ruled out [67]. We also noted that, although only individuals residing in Germany at the time of the survey participated in the study, the possibility that they had been previously socialized in other countries cannot be excluded. It is not clear whether they had undertaken the body modifications in Germany.

For the German-speaking sample in the present study, we used the German version of the Uniqueness scale, the NfU-G [49], and further examined extreme body modifications. To the best of our knowledge, most previous studies used the Uniqueness Scale developed by Snyder and Fromkin [38] for their non-German samples [15, 19, 20, 42, 43], the four-item SANU scale developed by Lynn and Harris [45, 46, 70, 71], or an interpretative phenomenological analysis following a semi-structured interview [44]. Previous studies did not include extreme body modifications, nor did they split NfU into its subcomponents.

Albeit considering NfU as a motivation for modification of the body and not vice versa is evident, a cross-sectional study design cannot determine whether NfU precedes the body modification or is its consequence. However, the causality is theoretically well-founded and can, therefore, be assumed in this study.

All of these issues result in possible desiderata for further research. A longitudinal study design could provide more information. Moreover, of major interest would be to investigate, if possible, the NfU of tattooed or pierced, or extreme-body-modified individuals in a comparative analysis. In other words, individuals having only one of the features should be investigated.

Despite these apparent limitations, this study has provided an understanding of apparent differences in the subcomponents of NfU for modifying the body. The research questions that were initially formulated could be answered: Tattooed, pierced, and extreme-body-modified individuals showed a greater manifestation of NfU than individuals without body modifications. Building on that, it seemed that individuals with tattoos took a social component into consideration while lacking concern regarding others’ reaction toward their tattoos although not wanting to cause affront. Pierced and extreme-body-modified individuals, contrarily, tended to display a propensity to actively flout rules and not worry about others’ opinions on their modifications. The study also showed that the number of body modifications increased depending on the extent of the expression of NfU. The results support the assumption that body modifications can be exploited to create self-expression or construct identities. The unique highlighting of the body can help achieve an improved perception of one’s uniqueness [72]. Therefore, body modifications can be an essential medium for developing unique identities by means of physical appearance.

## Supporting information

S1 Table Participants’ sociodemographic data. (DOCX)

S1 Forums List of forums and social media feeds focusing on body modifications used for data collection. (DOCX)

S1 File (DOCX)

## References

[1] Anderson R. Tattooing should be regulated. New England Journal of Medicine. 1992;326(3):207–207. 10.1056/NEJM199201163260318 1727564

[2] Borkenhagen A, Mirastschijski U, Petrowski K, Brähler E. Tattoos in der deutschen Bevölkerung–Prävalenzen, Soziodemografie und Gesundheitsorientierung. Bundesgesundheitsblatt—Gesundheitsforschung—Gesundheitsschutz. 2019;62(9):1077–1082. 10.1007/s00103-019-02999-7 31420716

[3] Tattoos & Piercing—Anzahl der Tattoos in Deutschland nach Alter im Jahr 2017 | Statista [Internet]. Statista. 2017 [cited 1 May 2020]. Available from: https://de.statista.com/statistik/daten/studie/719272/umfrage/umfrage-zur-anzahl-der-tattoos-in-deutschland-nach-alter/

[4] Armstrong ML. Career-oriented women with tattoos. Image: the Journal of Nursing Scholarship. 1991;23(4):215–220. 10.1111/j.1547-5069.1991.tb00674.x 1937518

[5] Armstrong ML, McConnell C. Tattooing in adolescents, more common than you think: the phenomenon and risks. Journal of School Nursing. 1994;10:22–29. 7. 8161874

[6] Armstrong ML, Roberts AE, Owen DC, Koch JR. Contemporary college students and body piercing. Journal of Adolescent Health. 2004;35(1):58–61. 10.1016/j.jadohealth.2003.08.012 15193575

[7] DeMello M. “Not just for bikers anymore”: popular representations of American tattooing. The Journal of Popular Culture. 1995;29(3):37–52. 10.1111/j.0022-3840.1995.00037.x

[8] DeMello M, Rubin G. Bodies of inscription. Durham: Duke University Press; 2000.

[9] Swami V, Harris AS. Body art (tattoos and piercings). In: Cash T, editor. Encyclopedia of Human Appearance and Body Image. Elsevier; 2012. pp. 58–65.

[10] Gump W. Modern induced skull deformity in adults. Neurosurgical Focus. 2010;29(6):E4. 10.3171/2010.10.FOCUS10203 21121718

[11] Kasten E, Wessel A. Piercings. In: Borkenhagen A, Stirn A, Brähler E, editors. Body Modification. MWV; 2014. pp. 21–39.

[12] Bone A, Ncube F, Nichols T, Noah ND. Body piercing in England: a survey of piercing at sites other than earlobe. BMJ. 2008;336(7658):1426–1428. 10.1136/bmj.39580.497176.25 18556275 PMC2432173

[13] Papameletiou D, Zenié A, Schwela D, Bäumler W. Risks and health effects from tattoos, body piercing and related practices. European Commission, Directorate General, Joint Research Centre; 2003.

[14] Piercing Siegmund-Schultze N. Unter die Haut: Körperschmuck mit Risiken. Deutsches Ärzteblatt. 2008;105(28–29): S. C-1297 ff.

[15] Tate JC, Shelton BL. Personality correlates of tattooing and body piercing in a college sample: The kids are alright. Personality and Individual Differences. 2008;45(4):281–285. 10.1016/j.paid.2008.04.011

[16] Pöhlmann K, Eismann E, Weidner K, Stirn A. Tätowierungen. In: Borkenhagen A, Stirn A, Brähler E, editors. Body Modification. MWV; 2014. pp. 1–20.

[17] Pausch NC, Garve R, Krey KF. Dento-orales Tuning–Schönheit im Mund. In: Borkenhagen A, Stirn A, Brähler E, editors. Body Modification. MWV; 2014. pp. 91–106.

[18] Schramme T. Should we prevent non-therapeutic mutilation and extreme body modification? Bioethics. 2007;22(1):8–15. 10.1111/j.1467-8519.2007.00566.x 18154584

[19] Tiggemann M, Golder F. Tattooing: An expression of uniqueness in the appearance domain. Body Image. 2006;3(4):309–315. 10.1016/j.bodyim.2006.09.002 18089234

[20] Tiggemann M, Hopkins LA. Tattoos and piercings: Bodily expressions of uniqueness?. Body Image. 2011;8(3):245–250. 10.1016/j.bodyim.2011.03.007 21561820

[21] Pöhlmann K, Joraschky P. Körperbild und Körperbildstörungen: Der Körper als gestaltbare Identitätskomponente. PiD—Psychotherapie im Dialog. 2006;7(2):191–195. 10.1055/s-2006-932635

[22] Greif J, Hewitt W, Armstrong ML. Tattooing and body piercing. Clinical Nursing Research. 1999;8(4):368–385. 10.1177/10547739922158368 10855104

[23] Millner VS, Eichold BH. Body Piercing and Tattooing Perspectives. Clinical Nursing Research. 2001;10(4):424–441. 10.1177/C10N4R7 11881952

[24] Wicklund RA, Gollwitzer PM. Symbolic self-completion. Routledge; 1982.

[25] Forbes GB. College students with tattoos and piercings: Motives, family experiences, personality factors, and perception by others. Psychological Reports. 2001;89(3):774–786. 10.2466/pr0.2001.89.3.774 11824749

[26] Wohlrab S, Stahl J, Kappeler PM. Modifying the body: Motivations for getting tattooed and pierced. Body Image. 2007;4(1):87–95. 10.1016/j.bodyim.2006.12.001 18089255

[27] Schildkrout E. Inscribing the body. Annual Review of Anthropology. 2004;33(1):319–344. 10.1146/annurev.anthro.33.070203.143947

[28] Stirn A. Body piercing: medical consequences and psychological motivations. The Lancet. 2003;361(9364):1205–1215. 10.1016/S0140-6736(03)12955-8 12686054

[29] Stirn A. Motivationen von Tätowierten und Gepiercten für ihre Körpermodifikationen. Zeitschrift für Klinische Psychologie, Psychiatrie und Psychotherapie. 2004;51:43–58.

[30] Vale V, Juno A. Modern primitives. San Francisco: Re/Search Publications; 1989.

[31] Atkinson M. Tattooing and Civilizing Processes: Body Modification as self-control. Canadian Review of Sociology/Revue canadienne de sociologie. 2004;41(2):125–146. 10.1111/j.1755-618x.2004.tb02173.x 15290832

[32] Laumann AE, Derick AJ. Tattoos and body piercings in the United States: A national data set. Journal of the American Academy of Dermatology. 2006;55(3):413–421. 10.1016/j.jaad.2006.03.026 16908345

[33] Nathanson C, Paulhus DL, Williams KM. Personality and misconduct correlates of body modification and other cultural deviance markers. Journal of Research in Personality. 2006;40(5):779–802. 10.1016/j.jrp.2005.09.002

[34] Sweeney SM. Tattoos: a review of tattoo practices and potential treatment options for removal. Current Opinion in Pediatrics. 2006;18(4):391–395. 10.1097/01.mop.0000236388.64333.cd 16914993

[35] Young M. Flesh journeys: Neo primitives and the contemporary rediscovery of radical body modification. Deviant Behavior. 2001;22(2):117–146. 10.1080/016396201750065018

[36] Winchel RM, Stanley M. Self-injurious behavior: a review of the behavior and biology of self- mutilation. American Journal of Psychiatry. 1991;148(3):306–317. 10.1176/ajp.148.3.306 1847025

[37] Turner BS. The possibility of primitiveness: Towards a sociology of body marks in cool societies. Body & Society. 1999;5(2–3):39–50. 10.1177/1357034X99005002003

[38] Snyder CR, Fromkin HL. Abnormality as a positive characteristic: The development and validation of a scale measuring need for uniqueness. Journal of Abnormal Psychology. 1977;86(5):518–527. 10.1037/0021-843X.86.5.518

[39] Snyder CR, Fromkin HL. Uniqueness: The pursuit of difference. Plenum Press. 1980; 10.1007/978-1-4684-3659-4

[40] Jarymowicz M, Codol J-P. Self-others similarity perception: Striving for diversity from other people. Polish Psychological Bulletin. 1979;10(1), 41–48.

[41] Imhoff R, Erb H-P. What motivates nonconformity? Uniqueness seeking blocks majority influence. Personality and Social Psychology Bulletin. 2009;35(3):309–320. 10.1177/0146167208328166 19098256

[42] Swami V. Written on the body? Individual differences between British adults who do and do not obtain a first tattoo. Scandinavian Journal of Psychology. 2012;53(5):407–412. 10.1111/j.1467-9450.2012.00960.x 22830568

[43] Swami V, Pietschnig J, Bertl B, Nader IW, Stieger S, Voracek M. Personality differences between tattooed and non-tattooed individuals. Psychological Reports. 2012;111(1):97–106. 10.2466/09.07.21.PR0.111.4.97-106 23045851

[44] Kalanj-Mizzi SA, Snell TL, Simmonds JG. Motivations for multiple tattoo acquisition: An interpretative phenomenological analysis. Advances in Mental Health. 2018;17(2):196–213. 10.1080/18387357.2018.1537127

[45] Owen DC, Armstrong ML, Koch JR, Roberts AE. College students with body art: Well-being or high-risk behavior? Journal of Psychosocial Nursing and Mental Health Services. 2013;51(10):20–28. 10.3928/02793695-20130731-03 23938068

[46] Lynn M, Harris J. Individual differences in the pursuit of self-uniqueness through consumption. Journal of Applied Social Psychology. 1997;27(21):1861–1883. 10.1111/j.1559-1816.1997.tb01629.x

[47] Camps FD, Bellut A, de Kernier N. La suspension corporelle: une clinique de l’extrême [Body suspension: a clinic of extremes]. Annales Médico-psychologiques, revue psychiatrique. 2015;17(8):688–694. 10.1016/j.amp.2015.04.013

[48] Kaatz M, Elsner P, Bauer A. Body-modifying concepts and dermatologic problems: Tattooing and piercing. Clinics in Dermatology. 2008;26(1):35–44. 10.1016/j.clindermatol.2007.10.004 18280903

[49] Schumpe BM, Herzberg PY, Erb H-P. Assessing the need for uniqueness: Validation of the German NfU-G scale. Personality and Individual Differences. 2016;90:231–237. 10.1016/j.paid.2015.11.012

[50] Faul F, Erdfelder E, Buchner A, Lang A. Statistical power analyses using G*Power 3.1: Tests for correlation and regression analyses. Behavior Research Methods. 2009;41(4):1149–1160. 10.3758/BRM.41.4.1149 19897823

[51] Cohen J. Statistical power analysis for the behavioural sciences. Hove: Academic Press; 1988.

[52] Field A. Discovering statistics using IBM SPSS statistics. Sage; 2018.

[53] Ellis PD. The essential guide to effect sizes: Statistical power, meta-analysis, and the interpretation of research results. Cambridge, New York: Cambridge University Press. 2010. 10.1017/CBO9780511761676

[54] Lalot F, Cantarella M., Zerhouni O, Joly E, Quiamzade A, Falomir-Pichastor JM, et al. Assessing private and public Need for Uniqueness: Validation of French versions of the Need for Uniqueness (NfU) and Self-Attributed Need for Uniqueness (SANU) scales. Journal of Personality Assessment. 2019; 101(3): 294–304. 10.1080/00223891.2017.1394867 29236555

[55] Stirn A, Brähler E, Hinz A. Prävalenz, Soziodemografie, mentale Gesundheit und Geschlechtsunterschiede bei Piercing und Tattoo. PPmP—Psychotherapie Psychosomatik Medizinische Psychologie. 2006;56(11):445–449. 10.1055/s-2006-951817 17091447

[56] Kunda Z. The case for motivated reasoning. Psychological Bulletin. 1990;108(3):480–98. 10.1037/0033-2909.108.3.480 2270237

[57] Swami V, Tran US, Kuhlmann T, Stieger S, Gaughan H, Voracek M. More similar than different: Tattooed adults are only slightly more impulsive and willing to take risks than non-tattooed adults. Personality and Individual Differences. 2016;88:40–44. 10.1016/j.paid.2015.08.054

[58] Armstrong ML. Adolescent tattoos: Educating vs. pontificating. Pediatric Nursing. 1995;21(6):561–564. 8700613

[59] Armstrong ML, Murphy KP. Tattooing: another adolescent risk behavior warranting health education. Applied Nursing Research. 1997;10(4):181–189. 10.1016/s0897-1897(97)80560-5 9419914

[60] Frederick CM, Bradley KA. A Different kind of normal? Psychological and motivational characteristics of young adult tattooers and body piercers. North American Journal of Psychology. 2000;2(2):380–393.

[61] Martin A. On Teenagers and Tattoos. Journal of the American Academy of Child & Adolescent Psychiatry. 1997;36(6):860–861. 10.1097/00004583-199706000-00025 9183143

[62] Lewin K. Dynamic theory of personality. McGraw-Hill; 1935.

[63] Barnlund DC. Public and private self in Japan and the United States. Simul Press; 1975.

[64] Roberti JW, Storch EA. Psychosocial Adjustment of College Students with Tattoos and Piercings. Journal of College Counseling. 2005;8(1):14–19. 10.1002/j.2161-1882.2005.tb00068.x

[65] Kim HS, Drolet A. Choice and self-expression: A cultural analysis of variety-seeking. Journal of Personality and Social Psychology. 2003;85(2):373–382. 10.1037/0022-3514.85.2.373 12916577

[66] Laatz W. Empirische Methoden. Thun: H. Deutsch; 1993.

[67] Schmidt-Atzert L, Amelang M. Psychologische Diagnostik. Springer; 2012.

[68] Furnham A. Response bias, social desirability and dissimulation. Personality and Individual Differences. 1986;7(3):385–400. 10.1016/0191-8869(86)90014-0

[69] Rosenthal R, Fode KL. The effect of experimenter bias on the performance of the albino rat. Behavioral Science. 2007;8(3):183–189. 10.1002/bs.3830080302

[70] Nelius T, Armstrong ML, Young C, Roberts AE, Hogan L, Rinard K. Prevalence and implications of genital tattoos: A site not forgotten. British Journal of Medical Practitioners. 2014;7(4).73.

[71] Swami V. Marked for life? A prospective study of tattoos on appearance anxiety and dissatisfaction, perceptions of uniqueness, and self-esteem. Body Image. 2011;8(3):237–244. 10.1016/j.bodyim.2011.04.005 21641893

[72] Huxley C, Grogan S. Tattooing, piercing, healthy behaviours and health value. Journal of Health Psychology. 2005;10(6): 831–841. 10.1177/1359105305057317 16176960

