# Supplementary material for: When personality gets under the skin: Need for uniqueness and body modifications
Source: PLoS One. 2021 Mar 3;16(3):e0245158. doi: 10.1371/journal.pone.0245158 (PMC7928480; doi:10.1371/journal.pone.0245158)
Supplement: S1 Table — (DOCX) [file pone.0245158.s001.docx]

| **S1 Table. Participants’ sociodemographic data** | | | | | | | | | | | |
| --- | --- | --- | --- | --- | --- | --- | --- | --- | --- | --- | --- |
| Variable | | | N, (*n*) | *%* | *Mean* | *SD* | *Median* | *Min* | *Max* | *IQR* |  |
| Age | | | 312 | 100 | 28.58 | 11.57 | 23.00 | 18 | 66 | 12 |  |
| Gender | | Female | 194 | 62.18 |  | | | | | |  |
|  |  | Male | 117 | 37.50 |  |  |  |  |  |  |  |
|  |  | Nonbinary | 1 | 0.32 |  |  |  |  |  |  |  |
| Residence | | Hamburg | 99 | 31.73 |  | | | | | |  |
|  |  | Bremen | 38 | 12.18 |  |  |  |  |  |  |  |
|  |  | Other | 175 | 56.09 |  |  |  |  |  |  |  |
| Birthplace | | Germany | 301 | 96.47 |  | | | | | |  |
|  |  | Other | 11 | 3.53 |  |  |  |  |  |  |  |
| Marital status | | Single | 147 | 47.12 |  | | | | | |  |
|  |  | Partnership | 85 | 27.24 |  |  |  |  |  |  |  |
|  |  | Married | 71 | 22.76 |  |  |  |  |  |  |  |
|  |  | Other | 9 | 2.88 |  |  |  |  |  |  |  |
| Highest education | | Lower secondary education (min. 9 years) | 4 | 1.28 |  | | | | | |  |
|  |  | Upper secondary education (min. 10 years) | 46 | 14.74 |  |  |  |  |  |  |  |
|  |  | Abitur (min. 12 years) | 162 | 51.92 |  |  |  |  |  |  |  |
|  |  | Advanced technical college certificate | 36 | 11.54 |  |  |  |  |  |  |  |
|  |  | University degree | 55 | 17.63 |  |  |  |  |  |  |  |
|  |  | Other | 9 | 2.88 |  |  |  |  |  |  |  |
| Current employment | | Unemployed | 1 | 0.32 |  | | | | | |  |
|  |  | Pupils | 2 | 0.64 |  |  |  |  |  |  |  |
|  |  | Students | 144 | 46.15 |  |  |  |  |  |  |  |
|  |  | Trainee | 24 | 7.69 |  |  |  |  |  |  |  |
|  |  | Part-time employed | 38 | 12.18 |  |  |  |  |  |  |  |
|  |  | Full-time employed | 79 | 25.32 |  |  |  |  |  |  |  |
|  |  | Self-employed | 13 | 4.17 |  |  |  |  |  |  |  |
|  |  | Unemployable | 1 | 0.32 |  |  |  |  |  |  |  |
|  |  | Other | 10 | 3.21 |  |  |  |  |  |  |  |
| Chronic physical illness | | No | 278 | 89.10 |  | | | | | |  |
|  |  | Yes | 34 | 10.90 |  |  |  |  |  |  |  |
| Psychiatric illness | | No | 257 | 82.37 |  | | | | | |  |
|  |  | Yes | 55 | 17.63 |  |  |  |  |  |  |  |
| Existing trauma | | No | 206 | 66.00 |  | | | | | |  |
|  |  | Yes | 106 | 34.00 |  |  |  |  |  |  |  |
| Tobacco consume | | No | 250 | 80.13 |  | | | | | |  |
|  |  | Yes | 62 | 19.87 |  |  |  |  |  |  |  |
| Alcohol consume | | No | 70 | 22.44 |  | | | | | |  |
|  |  | Yes | 242 | 77.56 |  |  |  |  |  |  |  |
| Illegal drug consumes | | No | 178 | 57.05 |  | | | | | |  |
|  |  | Yes | 134 | 42.95 |  |  |  |  |  |  |  |
| Body modification types | | Only tattoos | 32 | 10.26 |  | | | | | |  |
|  |  | Only piercings | 32 | 10.26 |  |  |  |  |  |  |  |
|  |  | Only extreme body modifications | 1 | 0.32 |  |  |  |  |  |  |  |
|  |  | All types of body modification | 21 | 6.73 |  |  |  |  |  |  |  |
|  |  | None | 174 | 55.77 |  |  |  |  |  |  |  |
|  |  | Only Tattoos & Piercings | 49 | 15.70 |  |  |  |  |  |  |  |
|  |  | Only Tattoos and Extreme body modifications | 1 | 0.32 |  |  |  |  |  |  |  |
|  |  | Only Piercings & Extreme body modifications | 2 | 0.64 |  |  |  |  |  |  |  |
| Percentage tattoo coverage | | | 103 | 33.01 | 10.50 | 13.55 | 5.00 | 1 | 80 | 13 |  |
| Total visibility | No | | 10 | 7.25 |  | | | | | |  |
|  | Yes | | 128 | 92.75 |  |  |  |  |  |  |  |

N, sample size; *n*, subsample size; *%*, percentage of participants, *SD*, standard deviation; *Min*, minimum; *Max*, maximum; IQR, interquartile range.
