# Supplementary material for: When personality gets under the skin: Need for uniqueness and body modifications
Source: PLoS One. 2021 Mar 3;16(3):e0245158. doi: 10.1371/journal.pone.0245158 (PMC7928480; doi:10.1371/journal.pone.0245158)
Supplement: S1 Forums — (DOCX) [file pone.0245158.s002.docx]

**S1 Forums. List of forums and social media feeds focusing on body modifications used for data collection**

- https://www.facebook.com/groups/523464631087424/
- https://www.facebook.com/Body.Modification.org/
- https://www.facebook.com/groups/609441015793444/
- https://www.tattooscout.de/forum/
