# Supplementary material for: When personality gets under the skin: Need for uniqueness and body modifications
Source: PLoS One. 2021 Mar 3;16(3):e0245158. doi: 10.1371/journal.pone.0245158 (PMC7928480; doi:10.1371/journal.pone.0245158)
Supplement: S1 File — (DOCX) [file pone.0245158.s003.docx]

**S1 File.**

We used the very conservative and robust Bonferroni correction in this study. A Holm-Bonferroni method and a Bayesian T-test were applied and resulted in similar results. The results can be provided by the authors upon request.
